# Gait change in tongue movement

**DOI:** 10.1038/s41598-021-96139-4

**Published:** 2021-08-16

**Authors:** Donald Derrick, Bryan Gick

**Affiliations:** 1 New Zealand Institute of Language, Brain, and Behaviour, University of Canterbury, Christchurch, 8041 New Zealand; 2 Department of Linguistics, University of British Columbia, Vancouver, BC V6T 1Z4 Canada; 3 Haskins Laboratories, New Haven, CT 06511-6695 USA

**Keywords:** Biophysics, Evolution, Physiology

## Abstract

During locomotion, humans switch *gaits* from walking to running, and horses from walking to trotting to cantering to galloping, as they increase their movement rate. It is unknown whether gait change leading to a wider movement rate range is limited to locomotive-type behaviours, or instead is a general property of any rate-varying motor system. The tongue during speech provides a motor system that can address this gap. In controlled speech experiments, using phrases containing complex tongue-movement sequences, we demonstrate distinct gaits in tongue movement at different speech rates. As speakers widen their tongue-front displacement range, they gain access to wider speech-rate ranges. At the widest displacement ranges, speakers also produce categorically different patterns for their slowest and fastest speech. Speakers with the narrowest tongue-front displacement ranges show one stable speech-gait pattern, and speakers with widest ranges show two. Critical fluctuation analysis of tongue motion over the time-course of speech revealed these speakers used greater effort at the beginning of phrases—such end-state-comfort effects indicate speech planning. Based on these findings, we expect that categorical motion solutions may emerge in any motor system, providing that system with access to wider movement-rate ranges.

## Introduction

The study of gait and gait-change, traditionally limited to human and vertebrate animal locomotion, has fascinated philosophers since ancient times^1^. Understanding the relationship between human step, cadence, and walking speed required Galileo’s bridging of experimental research and deductive reasoning, Descartes’ coordinate system, and De Homine’s more complete human physiology, as well as the invention of the stopwatch and the telescope^2,3^. The task of measuring detailed gait motion accelerated the invention of sequential^4,5^ and overlayed^6^ photography, motion picture technology, force plate systems, and motion capture systems^3^. Various conflicting explanations have been proposed to account for why gait changes as locomotion speed increases^7^. These include ones based on metabolic efficiency^8^, mechanical load^9^, mechanical efficiency^10^, and cognitive factors^11^. Researchers have sought answers by expanding the domain of gait research to include the effects of uneven surfaces and aging on gait^12^, as well as gait-like behaviour in swimming^13^, flying^14^, and bimanual finger and hand coordination^15,16^. These studies have generally been limited to motor systems with skeletal or rigid support structures, driven by innate central pattern generators^17^, performing locomotive or entrainment tasks.

Some researchers have sought evidence of gait-like behaviour in limb and finger rotation. Kelso and colleagues’ interlimb^15^ and bimanual^16^ coordination research shows that as people move limbs or fingers cyclically and in anti-phase to each other, they abruptly switch to in-phase as their rate of motion increases. Similar change in motion occurs within a single person’s body^15,16^, in coordination with two people^18^, or in coordination with external stimuli^19^. These patterns are reminiscent of the out-of-phase leg motion patterns in a horse’s trot, as compared to the in-phase motion of a gallop. Such observations can be taken as evidence of a phase transition as rate increases.

In a rate-varying speech paradigm, Tuller and Kelso^20^, and later de Jong et al.^21^, showed that as speech rate increases, speakers producing sequences of ‘ip’ sound to perceivers as if they are saying ‘pi’. This may be because the normal opening and closing of the vocal folds during vowel production destabilizes at increasingly high speeds until the vocal folds vibrate continuously, leaving only the lips and jaw opening and closing as they continue the cycle of ‘p’ production. This change affects how people hear the syllable. When producing a sequence of identical ‘pi’ syllables at a comfortable rate, English speakers will typically insert a glottal stop (the catch in the throat heard in the middle of ‘uh-oh’) before the vowel to indicate the onset of each syllable. Without this glottal stop preceding the ‘i’ for each ‘ip’ syllable, listeners reinterpret the ‘p’ as the syllable onset. While this is clearly an example of rate-varying speech behavior, it is not a strategic shift allowing the system to succeed under a wider range of movement rates—i.e., the change in behavior does not appear to convey a benefit. Rather, these experiments document a deterioration of glottal performance under stress such that speakers can no longer successfully produce the intended distinction between syllables.

Here we investigate a beneficial rate-varying behaviour in a non-innate, non-locomotive biological system that does not rely on rigid skeletal support. The speaking human tongue provides such a system. The neural control of locomotive gait is innate^22^, and spinal^23^, whereas tongue movement in speech is learned and controlled by the brain. Speech is also phylogenetically young, drawing on older neural substrates that evolved for suckling, swallowing and chewing^24^. During speech, the goals of the tongue’s movements are to produce communicative sound. The tongue is small compared to our limbs, and non-weight-bearing, so it is unlikely to be constrained by metabolic efficiency or mechanical load. And unlike legs or fingers^16,18,19^, the tongue is a minimally unconstrained flexible muscular hydrostatic system, more similar to a tentacle or an elephant trunk^25^. The tongue’s only direct attachments to the skeletal system is at its base via the mandible and the “floating” hyoid bone.

Yet similar to locomotion, speech is highly rate-varying; the same person can say the same utterance quickly or slowly. Patterns of vocal tract behaviour in slow and fast speech are known to differ tremendously from each other^26^, as do their neurophysiological control mechanisms^27^. These differences can have profound effects on production and perception. Observations such as these have made researchers draw analogies between speech and locomotive gait: Speech simulation research^28^, corroborated by a study of reaction latency^29^, predicts that speech should be associated with differing fast and non-fast speech ‘gaits’. However, until now, such speech gaits have not been directly observed in the speech articulators. Observing gait-change-like behaviour in tongue motion during speech would show that gait change is not dependent on the neurophysiological structures associated with locomotive gait, but is instead an emergent property of motor systems operating under rate-varying conditions.

The tongue tip is the most flexible and freely mobile part of the tongue^30^. Observing gait change in speech is possible because of North American English (NAE) *flap* movements. Flaps  are produced by flicking or tapping the tongue tip against the roof of the mouth. These flap movements, which produce sounds like the ‘dd’ in ‘ladder’, or the two ‘d/t’ sounds in ‘editor’, have multiple categorically distinct movement variants^31^ that can be distinguished based on tongue movement direction, i.e., whether the tongue tip moves up, down, or across to contact the hard palate. Research shows that patterns of tongue motions during sequences of vowels and flaps are particularly variable and unstable^32,33^. For instance, some North American English speakers moved their tongues in upwards of four categorically different patterns during otherwise identical repetitions of the word ‘murder’^31^. These tongue movement sequences are some of the few that are big enough to measure using available technology. As a result, they are ideal for testing the hypothesis that people employ different categorical motion strategies (analogous to walking vs. running) across different speech rates. Specifically, we predict that speakers can shift tongue motion patterns in flap sequences as they increase their speech rate, and by so doing, gain access to wider speech-rate ranges.

In addition, to solidify a claim of gait-change, we need to identify evidence of planned gaits that can disambiguate them from unstable phase transitions between those gaits. Historically, such has been demonstrated by showing that the transition between gaits involve critical fluctuations at some boundary between two more stable and distinct phases of motion, as Kelso shows in his bimanual coordination research^16^. Using another example, a person who is running and then slows down often transitions from a smooth running motion to a couple of jerky steps and then into a smooth walking motion. The smooth walking and running represent two different phase states, and the stumbling between the two shows critical fluctuations. The speed at which people transition differs based on whether the person is speeding up from a walk, or slowing down from a run—this difference is evidence of hysteresis^10^. However, we cannot use critical fluctuations, critical slowing, or hysteresis to observe the speeds at which gait transitions take place when speakers progressively speed up or slow down. This is because our experimental paradigm was designed to identify speech motion strategies that provide access to wider speech-rate ranges, rather than identify particular patterns of articulator motion breakdown when slowly speeding up or slowing down speech.

Instead, we can observe the time course of each utterance and apply a well-known measure of motor planning to identify stable gaits and distinguish them from gait (phase) transition. We do this by first tracking critical fluctuations during the time course of each utterance. Using recent innovations in applied mathematics, we overcome the requirement for large amounts of sequential data to identify critical fluctuations required by “Pointwise Correlation Dimension (PD2^34,35^), the Local Largest Lyapunov exponent (LLLE^35–38^), or the Entropy Rates^37,39^. This measure of critical fluctuation requires a window of only 7 data points in a time series to work^39^. Doing so is useful because critical fluctuations provide a quantitative measure of effort in articulation—an idea as intuitive as recognizing how much effort is required to slow down from a run to a walk.

By measuring critical fluctuations during particular portions of speech, we can identify whether the speaker put the most effort in the beginning, middle, or end of one of these flap sequences (e.g. ‘editor’). Putting more effort in to the beginning of a complex utterance so that less effort is required at the end is an example of the *end-state-comfort* effect^40^. End-state-comfort effects are themselves a well-known demonstration that a motor system is using previous information and experiences to plan the next course of events^40^. Researchers have used end-state comfort effects to establish a relationship between cognition and biomechanics^41^. Researchers have also expanded the research and theoretical models to other animals^42,43^, arguing for the evolutionary roots of motor planning. We have also previously used end-state-comfort as evidence for speech planning in flap sequences^33^. With this information, we can then compare the timings of higher and lower critical fluctuation against a speaker’s ability to have a wider range of tongue motion displacement.

To reiterate, we predict that speakers can shift tongue motion patterns in flap sequences as they increase their speech rate, and by so doing, gain access to wider speech-rate ranges. At the widest of tongue motion ranges, these motion patterns may reveal categorical differences between slower and faster speech–gait-changes. We predict these gaits will be more planned and stable than tongue motion patterns that stretch the stereotypical gait pattern for slower and faster speech. As a result, we expect participants that use one stable gait per token type will produce speech with a narrow speech-rate range, and demonstrate end-state-comfort effects. Participants who use two stable gaits per token type will produce speech with a wide speech-rate range, and also demonstrate end-state-comfort effects.

## Methods

### Declaration

The University of Canterbury’s Human Ethics Committee (HEC) approved ethics for this study (HEC 2012/19). All experiments were performed in accordance with the relevant named guidelines, regulations, and agreed-upon procedures listed in the HEC 2012/19 document. Each participant provided informed consent before participating in the experiments. Participants were compensated with $40 New Zealand Dollars worth of local Westfield mall vouchers.

### Participants

We recorded 11 participants (9 female and 2 male). (Note: Because articulometry experiments are long and demanding, worldwide median participant counts are small (5 as of 2020^44^). All but one of the participants were native monolingual North American English (NAE) speakers, and the other was a native bilingual NAE and French speaker. Participants reported normal hearing following the Nobel^45^ paradigm, where participants are asked about any difficulty hearing, any difficulty following television programs at a socially acceptable volume, and their ability to converse in large groups or noisy environments.

### Materials

Setup included an NDI Wave EMA machine with 100 Hz temporal resolution and 16 five degrees-of-freedom (5D) sensor ports. Setup also included a General Electric Logiq E 2012 ultrasound machine with a 8C-RS wide-band micro-convex array 12 $\times$ 22 mm, 4–10 megahertz imaging frequency transducer. Audio was collected using a USB Pre 2 pre-amplifier connected to a Sennheiser MKH-416 short shotgun microphone mounted to a Manfrotto “magic arm” for directional control. Ultrasound data were captured using an Epiphan VGA2USB Pro frame grabber connected to a MacBook pro (late-2013) with a solid-state drive. The USB-Pre 2 audio output and NDI wave machine were connected to a Windows 7 desktop computer with the NDI Wavefront control and capture software installed. This setup allows simultaneous ultrasound, EMA, and audio recording of participants. In this study, the ultrasound measurements were used for visual confirmation of tongue movements only.

### Stimuli

We selected eight one- or two-word utterances, or *token types*, with double-flap sequences (e.g. ‘auditor’), and embedded them in carrier phrases that have no adjacent tongue motion-generating consonants (e.g. ‘We have **auditor** books’). The stimuli are all listed in Table 1. Stimuli were chosen to allow for a variety of surrounding vowel contexts, while simultaneously keeping the experiment short enough to allow the equipment to work effectively.

The phrase structures we used were designed to ensure that speakers would place primary stress on the syllable before the first flap, a context in which speakers are most likely to produce flap sequences^46^. We introduced different speech rates by having participants hear reiterant speech (e.g. ‘ma ma **ma** ma ma ma’) produced at one of five different speech rates (3–7 syllables per second). In our experiment, we had participants listen to this reiterent speech, and then read one of the eight phrases displayed on a computer screen at that reiterent speech rate to the best of their ability. Each example was randomly presented as 40 phrases per block, with 10 blocks in total, such that the entire task took 45 min to complete.

**Table 1:** Experiment stimuli list.

	Token type in carrier	Token type
1	“We may edit a book”	“Edit a”
2	“We may audit a book”	“Audit a”
3	“We have auditor books”	“Auditor”
4	“We have editor books”	“Editor”
5	“We have Saturday books”	“Saturday”
6	“We have bettered a book”	“Bettered a”
7	“We have worded her books”	“Worded her”
8	“We have herded her books”	“Herded her”

### Setup and procedure

After completing initial screening, each participant was seated in a comfortable chair and heard a detailed description of the experimental procedure. An ultrasound transducer was held in place beneath the chin using a soft, non-metallic stabilizer^47^, allowing participants’ tongue movements to be recorded using ultrasound. The ultrasound measurements were used for visual confirmation of tongue movements, but were otherwise not included in the analysis. Five-dimensional (5D) electromagnetic articulometry (EMA) sensors were taped to the skin over the mastoid processes behind the ears and the nasion. Sensors were then taped and glued midsagitally to the upper and lower lips on the skin next to the vermillion border using Epiglu. One sensor was then glued to the *lower incisor*, and three to the tongue: One approximately 1 cm away from the *tongue tip*, one at the back or *tongue dorsum*, just avoiding the gag reflex, and one in between the two or *tongue body*. Tongue sensors were then coated in Ketac, a two-part epoxy cement normally used in dental implants. Both the Epiglu and Ketac are slowly broken down by saliva, allowing about 1 h of experiment time.

Once sensors were connected, the MKH-416 short shotgun microphone attached to a Manfrotto magic arm was placed on the opposite side of the head from the NDI wave electric field generator. The microphone was far enough away to avoid electro-magnetic interference with the NDI sensors, but close enough to reduce the acoustic interference from the many machine fans used to cool equipment during the recordings. The NDI wave recordings were captured at 100 cycles per second (Hz), and the audio recordings were synchronously captured at 22,050 Hz using 16 bit pulse-code-modulation (a standard .wav file format).

Once the setup was complete, participants read 10 blocks each containing the 8 sentences in Table 1, at 5 different speech rates, presented on a computer using Psychopy2^48^. We induced different speech rates by having participants hear reiterant speech (e.g. ‘ma ma **ma** ma ma ma’) produced at one of five different speech rates (3, 4, 5, 6, or 7 syllables per second) before being asked to read the relevant phrase at the preceding reiterant speech rate. Within each block, sentences and speech rates were randomly presented. Participants read sentences at the reiterant speech rate as instructed and to the best of their ability. In the event of sensor detachment, the area around the sensor was quickly dried with a paper towel, and the sensor was reattached with Epiglu only, within 1 mm of the original attachment point. No sensor was reattached a second time.

Once the experiment was complete, the participant was asked to hold a protractor between their teeth with the flat end against the corners of the mouth, and three (3) 10-s recordings of the occlusal (bite) plane were recorded. Setup took between 30 and 45 min; recording took about 45 min; recording of the occlusal plane, palate, and head rotation took no more than 10 min; and removal of sensors took 5 min. The entire process was typically completed within 2 h.

### Data processing

EMA data were loaded from NDI-wave data files, and smoothed with a discrete cosign transform technique that effectively low-pass-filters the data and restores missing samples using an all-in-one process^49,50^. This process was implemented through MVIEW^51^. Data were then rotated to an idealized flat (transverse cut) occlusal plane with the tongue tip facing forward. This was accomplished using the recorded occlusal plane and the recorded planar triangle between the nasion and two mastoid processes, allowing all of the participants’ data to be rotated and translated to a common analysis space. Tongue palate traces were generated using the highest tongue sensor positions along the midsagittal plane, correcting for extreme outliers.

Acoustic recordings were transcribed, isolating the phrases in one transcription tier, the vowel-flap-vowel-flap-vowel sequences under analysis in a second tier, and the two flap contacts in a third tier. Flap contacts were identified by the acoustic amplitude dip^46^, or by ear if the flap was approximated enough to not have an amplitude dip (such approximants were rare, accounting for less than 10% of the data).

**Figure 1:**
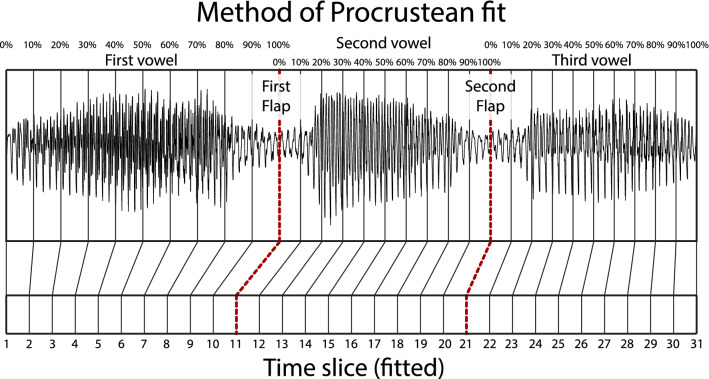
Procrustean fit time slices for ‘editor’, as spoken by participant 3, block 10, at 3 syllables/s.

In order to compare different speech rates, the acoustic and vocal tract movement information was subdivided into 31 time slices: Eleven (11) from the onset of the first vowel to the point of lowest acoustic intensity of the first flap, 10 more from that point to the point of the lowest acoustic intensity of the second flap, and from there, 10 more to the end of the following vowel. The entire time span constitutes the duration of each token type. These Procrustean fits allowed comparison of tongue motion and acoustic information at the same relative timing regardless of speech rate, and an example is illustrated in Fig. 1.

Acoustic cues were chosen because our previous research showed that flaps in English can be categorized in at least four patterns. Two of them, *alveolar-taps* and *post-alveolar taps*, involve tongue tip and blade motion towards the teeth or hard palate, making light contact, and moving away again. Two others, *up-flaps* and *down-flaps*, involve the tongue making tangential contact with the teeth or hard palate^31^. These subphonemic difference mean that it is impossible to identify flap contact through articulatory gesture identification tools such as FindGest^51^. However, there is almost always a direct and simultaneous relationship between the point of lowest amplitude in the acoustic signal and the timing of tongue to palate/teeth contact during flap production^46^. This makes acoustic cues the most suitable method of isolating the underlying articulatory motion patterns for this dataset.

### Visualization

Movement data from these Procrustean fits were visualized on millimetre-grid graphs. The graphs show the palate and position traces of the tongue tip, tongue body, tongue dorsum, lower incisor, upper lip, and lower lip throughout token production for each reiterant speech rate from 3 to 7 syllables/s. These graphs were produced for each participant and token type, with movement traces averaged over all the blocks. Versions of this graph tracing each block separately were used to identify cases where EMA sensors became unglued from the participants’ tongues, or sensor wires had tiny breakages. These tokens were excluded from analysis. Lastly, visual comparison of the different speech-rate traces revealed a wide variety of tongue motion pattern differences between participants, token types, and speech rates.

### Analysis: displacement range and speech-rate range

In order to test the prediction that speakers can shift tongue motion patterns in flap sequences to gain access to wider speech-rate ranges, we needed to compare duration to tongue motion patterns. Duration was measured from the start of the first vowel to the end of the third vowel—the span shown in Figure 1. However, there were so many different tongue motion pattern differences between participants, token types, speech rates, and recording blocks that we needed two equations to linearize this complexity of motion. These equations convert all of the above complexity into a cumulative measure of tongue motion displacement that accounts for both the sum of distance of motion and the sum of angular displacement.

Equation (1) captures the sum of the linear distance of motion along the course of any given vocal tract sensor’s motion through the Procrustean fit.

1 $$\begin{aligned} D = \sum _{i=1}^{30} d _{\vec {(i,i+1)}}. \end{aligned}$$

Each vector is calculated from the linear displacement, in a Euclidean plane, of a vocal tract sensor between adjacent Procrustean time slices. The value *D* captures the sum of the 30 displacements *d* in each vector in order from $d_{\vec {(1,2)}}$ through to $d_{\vec {(30,31)}}$, where the subscript numbers represent the relevant position of the sensor at that Procrustean time slices.

Equation (2) captures the sum of the angular displacement along the course of any given vocal tract sensor’s motion through the Procrustean time slices.

2 $$\begin{aligned} \Theta = \sum _{i=1}^{29} |\theta _{\vec {(i,i+1)},\vec {(i+1,i+2)}}|. \end{aligned}$$

Each vector is the same as for Eq. (1). The value $\Theta$ captures the sum of the 29 angles ($\theta$) between each vector in order, from $|\theta _{\vec {(1,2)},\vec {(2,3)}}|$ through to $|\theta _{\vec {(29,30)},\vec {(30,31)}}|$, where the subscript numbers represent the relevant position of the sensor at that Procrustean time slice. The $\theta$ is always the smallest of the absolute value of two possibilities, and so is never more than $\pi$ radians.

**Figure 2:**
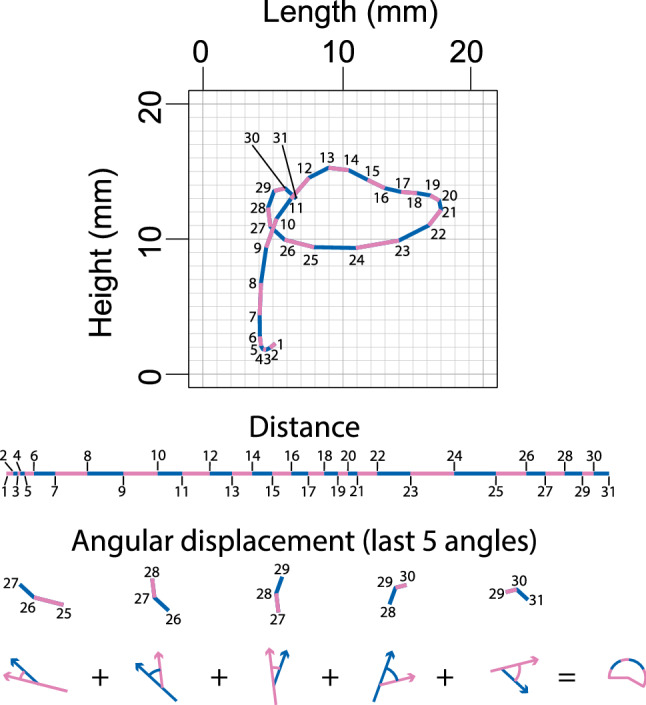
Illustration of the calculation process Eqs. (1) and (2). The top graph shows tongue tip motion for the average (mean) position of each instance of tongue-tip motion (facing right) for participant 9, token type ‘we have auditor books’, and reiterant speech at 3 syllables/s. The middle section shows a lining up of the path shown, giving the sum distance (for all values of i = 1–30 in Eq. 2). The bottom of the figure shows the visual process for calculating angular displacement for part of the tongue motion (showing only values of i = 25–29 in Eq. 1).

The process of computing each of these formulas is visualized in Fig. 2.

Because the measures for angular displacement (Eq. 2) are in radians, and distance (Eq. 1) are in millimetres, their scales are unrelated to each other. To resolve this issue, we applied z-scores to each, as seen in Eq. (3).

3 $$\begin{aligned} z = (x - \mu ) / \sigma , \end{aligned}$$

where (*x*) is the result from the relevant equation (Eq. 1 or 2), using the mean ($\mu$) divided by the standard deviation ($\sigma$). All z-scores were computed across the entire dataset (all token instances and all participants). This allows the results of both equations to be added together in a way that weights distance and angular displacement equally, giving us a measure of total *displacement*.

#### Displacement range

We then computed displacement range by comparing mean displacements for each participant (11 participants) and token type (8 types) produced at 3 syllables/s, and subtracting the mean displacements produced at 7 syllables/s. This provided us with displacement range data to compare to the average (mean) of the duration, grouped by participant (11 participants), token type (8 types), and reiterant speech rate (5 rates). We ran generalized linear mixed-effects models (GLMMs) as seen in Eq. (4).

4 $$\begin{aligned} Duration&\sim Displacement~Range~\times ~Reiterant~Speech~Rate~ \nonumber \\&\quad +(1~+~Displacement~Range~|~Participant) \end{aligned}$$

In this equation, written in R code^52^, *Duration* is equal to token utterance time, *Displacement Range* is the displacement range described above, *Reiterant Speech Rate* is one of 3–7 syllables/second, and *Participant* is the unique identifier for each research participant.

We ran this model for four measures of displacement range: (1) tongue tip only, (2) tongue tip and body (tongue front), (3) the whole tongue, and (4) the whole vocal tract. These four options were made as the tongue tip visually showed the most differences in displacement, followed by the tongue body, the tongue dorsum, and the lips/jaw which moved the least.

We then made ANOVA comparisons of the GLMMs ran with our four displacement ranges, and the tongue-front displacement range produced was the best fit. The model’s r^2^ was 0.816 for the fixed effects (r^2^m), and 0.892 when the random effect of participant variability was included (r^2^c). This comparison process allowed us to exclude sensors that did not add any statistically significant information to our analysis. Nevertheless, it must be recognized that the tongue tip and tongue body sensors naturally incorporate some jaw motion data as the tongue rides on the jaw.

#### Tongue-front displacement range

In more detail, the equation for tongue tip and body (tongue-front) displacement is seen in Eq. (5).

5 $$\begin{aligned} T = z((z(\Theta TT) + z(D TT) + z(\Theta TB) + z(D TB)). \end{aligned}$$

This equation shows z-scored tongue front [tongue tip (TT) and tongue body (TB)] distance (*d*) and angular displacements ($\theta$) summed together and then z-scored again so that the resulting sum displayed standard deviations in our graphs. We named this equation (Eq. 5) *tongue-front displacement*, and the displacement range—comparing mean displacements for each participant (11 participants) and token type (8 types) produced at 3 syllables/s, and subtracting the mean displacements produced at 7 syllables/s—is the *tongue-front displacement range*. This tongue-front displacement range, when graphed along with duration, allowed us to graph the relationship between tongue-front displacement range and speech-rate, revealing speech-rate range. This graph not only revealed speech-rate range, but allowed us to identify the shortest and longest displacement ranges and show the actual tongue motion patterns for both groups in a different figure.

#### Analysis: critical fluctuations

But in order to demonstrate that tongue-front displacement range is also associated with an increased likelihood of a speaker having two stable gaits instead of just one, we needed to compare critical fluctuations through the time-course of token production with tongue-front displacement range.

This equation for calculating critical fluctuation needed to work with a short-term time series—our 31 Procrustean time slices. One such equation is the fluctuation equation (F) from Schiepek and Strunk’s real-time monitoring of human change processes^39^. The fluctuation equation identifies positions of critical instability that indicate upcoming potential phase change by incorporating components of the 1st through 3rd derivative of the short-term time series. Differences in the patterns of phase change at different speech rates indicate that the changes in displacement also correspond to gait-change. This equation can work with as few as 7 data points. The fluctuation equation (Eq. 6) is as follows:

6 $$\begin{aligned} F = \dfrac{\sum \nolimits _{i=1}^{l} \dfrac{|x_{n_{k+1}}-x_{n_{k}}|}{(n_{k+1}-n_{k})}}{s(m-1)}. \end{aligned}$$

Unlike the formula in Schiepek et al.^39^, *n* is transformed by its relative position in time, as shown in the waveform in Fig. 1, such that the sum of the *n* values remains equal to *m*, but the ratio reflects the information required for maximum accuracy of the fluctuation calculation. The value $x_{n}$ is the *n*th number value in the time series. The value *k* indicates then number of points of return, that is, the number of times the time series values change direction. The value *i* represents the periods between points of return. The value *l* is the total number of periods within the window. The value *m* is the number of measurement points within a moving window, in our case 7. The value $m-1$ is the number of intervals between the measurement points, in our case 6. The value *s* = $x_{max} - x_{min}$; $x_{min}$ is the smallest value of the scale, in our case $-\pi$, and $x_{max}$ is the largest value of the scale, in our case $\pi$. This guarantees that the range for F is always a value between 0 and 1 (even with the *n* transformation above).

**Figure 3:**
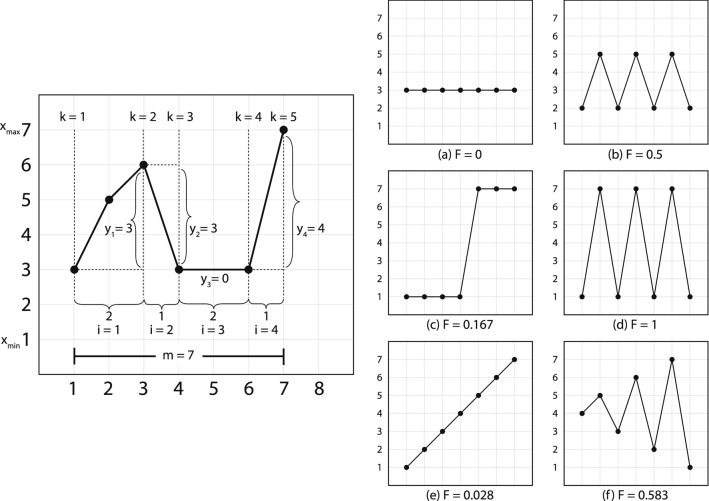
Calculation of F (Eq. 6). Left hand side shows a visualization of how to implement the algorithm. Right hand side shows six examples of the output of the algorithm. Note that the algorithm produces higher numbers from a combination of rate and amplitude of each change in direction. Image modified from Figs. 2 and 3 in Schiepek and Strunk^39^, used by permission following STM permissions guidelines.

Higher F-values indicate greater critical fluctuation, which itself corresponds to more production effort as compared to lower F-values. Lastly, F-values were calculated over two sets of data: (1) Tongue tip and (2) Tongue body $\{\theta _{\vec {1}}, \ldots , \theta _{\vec {30}}\}$, tracking through time slices of 7 vectors from $\{1, \ldots ,7\}$ through to $\{23, \ldots ,30\}$ such that these vector sets supply *x* in Eq. (6). The $\theta$ values were used because with a maximum range of $2\pi$ they meet the requirement for Eq. (6). Tongue tip and tongue body were used because they were the measurement sensors that carried the significant information for tongue-front displacement. These values were summed, and then divided by 2 to represent the tongue-front displacement fluctuations with a theoretical range of $0 \leqslant F \leqslant 1$. The algorithm is shown visually in Fig. 3, along with example paths and the fluctuation (F) value for each of them.

Next, and in order to make sense of this highly non-linear data, we ran a generalized additive mixed-effects model (GAMM) on the data shown in Eq. (7)^53,54^. GAMMs are extremely effective for the analysis of non-linear data, and are therefore highly suitable for the analysis of the critical fluctuations captured in Eq. (6).

7 $$\begin{aligned} gam(Fluctuation&\sim te(FTS,~TFd)~\nonumber \\&\quad + s(FTS,~TFd,~Participant,~bs =``fs'', m = 1)\nonumber \\&\quad + s(SPS,~Participant,~bs =``re'')~\nonumber \\&\quad + s(Token type,~Participant,~bs =``re''). \end{aligned}$$

Equation (7), written in R-code^52^, describes a generalized additive mixed-effects model, comparing *Fluctuation* based on tongue-front displacement (*TFd*) and the fluctuation time slice position (*FTS*), forming a 3-dimensional tensor (*te*) field [*te(FTS, TFd)*]. The random effects factor out *participant* variability in a 3-dimensional tensor field [*s(FTS, TFd, Participant, bs = “fs”, m = 1)*], as well as random-effect smooths for syllables per second [*s(SPS, Participant, bs = “re”)*], and token type [*s(Token type, Participant, bs = “re”)*]. In order to correct for autocorrelation effects, we ran the GAMM, calculated an estimate for a start value $\rho$ from that first run, and provided that $\rho$ to a second run of the GAMM model, along with an indicator identifying the first position of our time slices. This removes most of the autocorrelation, maximizing the accuracy of the resulting statistical model output.

Equation (7) produces an output that shows the relationship between critical fluctuation, token position, and tongue-front displacement range, highlighting regions of significant difference. And with these methods, we were able to identify whether tongue-front displacement range affected speech-rate range, and whether tongue-front displacement range had any influence on the timing slice positions of critical fluctuations.

## Results

**Figure 4:**
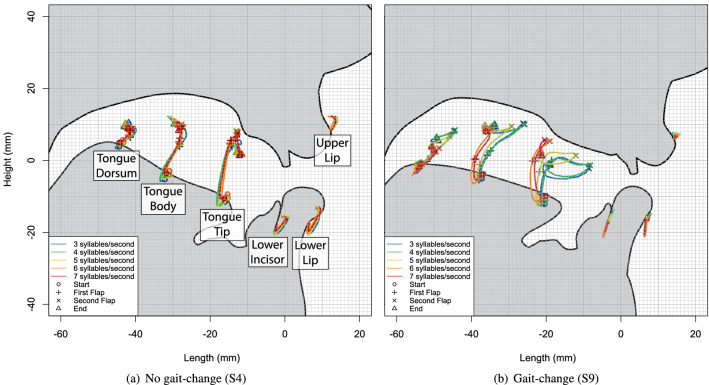
Vocal tract kinematic graphs comparing averaged tongue, jaw, and lip motions during productions of ‘auditor’ in the phrase ‘we have auditor books’ across five speech rates, showing: (**a**) S4: No categorical differences across speech rates; (**b**) S9: Clearly visible categorical differences across speech rates. Palate data were generated from palate estimation based on highest tongue positions in the dataset. Tongue, teeth, and face traces are based on initial tongue position and are otherwise provided for illustration purposes only.

We begin our results with examples to illustrate the positioning of tongue, jaw, and lip articulometry sensors within the vocal tract. Figure 4 shows two examples from the phrase ‘We have auditor books’, focusing on the token type ‘auditor’. Participant 4 shows almost no difference in the motion patterns between the slowest and fastest speech rates, as seen in Fig. 4a. In contrast, participant 9 shows a dramatic change in tongue-tip motion between the slowest and fastest rates, as seen in Fig. 4b.

### Results: displacement range and speech-rate range

Participants also demonstrated a wide range of ability to match the speech-rate range of the reiterant speech, with some participants and token types having a wide speech-rate range, and others having a narrower speech-rate range. This variation provided a basis for comparing speech-rate range to vocal tract articulator displacement (composed of the distance and angular displacement) differences for slow and fast speech.

**Figure 5:**
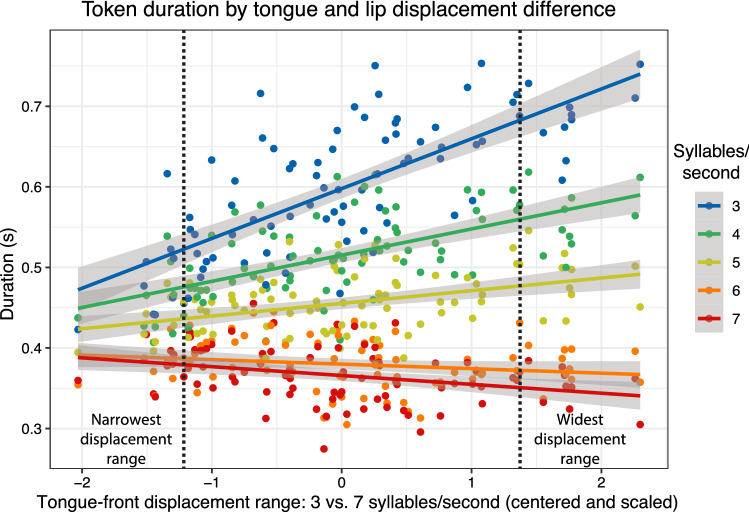
Comparison of tongue-front displacement range and speech rates between the responses to reiterant speech at 3 syllables/s and 7 syllables/s for all participants/token types. Values for the 10 participants/token types with the narrowest tongue-front displacement ranges are on the left side of the leftmost dashed-black line, and their tongue-tip motions are highlighted in Fig. 6a. Values for the 10 participants/token types with the widest tongue-front displacement ranges are on the right side of the rightmost dashed-black line, and their tongue-tip motions are highlighted in Fig. 6b.

This analysis is shown in Fig. 5. As described above, we compared the speech-rate range with tongue-front displacement range. Speech rates shown in the y-axis of Fig. 5. This information was placed along the tongue-front displacement range for each participant, as shown on the x-axis of Fig. 5. The mean durations for each participant, token type, and reiterant speech rate are shown in the colored dots in Fig. 5. A linear estimate fit was then shown for the relationship between the tongue-front displacement range and the speech rates produced in response to each reiterate speech rate. These form the colored lines in Fig. 5, and show show the predicted speech-rate range.

The trends seen in Fig. 5 are highly significant, as shown through GLMM analysis, and are shown in Table 2, and indicate a significant main effect of displacement, such that participants who had a wider tongue-front displacement range spoke more slowly than those who had a narrower tongue-front displacement range. There was also an expected main effect of reiterant speech rate, such that the slower the rate of reiterant speech, the slower the rate of speech for each participant. Lastly, there was a significant interaction between tongue-front displacement range and reiterant speech, such that participants who had a wider tongue-front displacement range also had a wider speech-rate range between each of the reiterant speech rates, except for the difference between 6 and 7 syllables/s, where the t-value difference is only 1.0, and therefore not significant.

**Table 2:** Results: generalized linear mixed-effects model (Eq. 4).

	Estimate	Std. Err.	DF	t-value	p-value
Displacement	0.060	0.006	14.6	10.1	< 0.001
4 syl/s vs. 3	− 0.083	0.005	407	− 16.4	< 0.001
5 syl/s vs. 3	− 0.142	0.005	407	− 28.3	< 0.001
6 syl/s vs. 3	− 0.218	0.005	407	− 43.2	< 0.001
7 syl/s vs. 3	− 0.232	0.005	407	− 46.0	< 0.001
Displacement $\times$ 4 syl/s vs. 3	− 0.029	0.005	407	− 5.82	< 0.001
Displacement $\times$ 5 syl/s vs. 3	− 0.046	0.005	407	− 9.14	< 0.001
Displacement $\times$ 6 syl/s vs. 3	− 0.067	0.005	407	− 13.4	< 0.001
Displacement $\times$ 7 syl/s vs. 3	− 0.073	0.005	407	− 14.4	< 0.001

**Figure 6:**
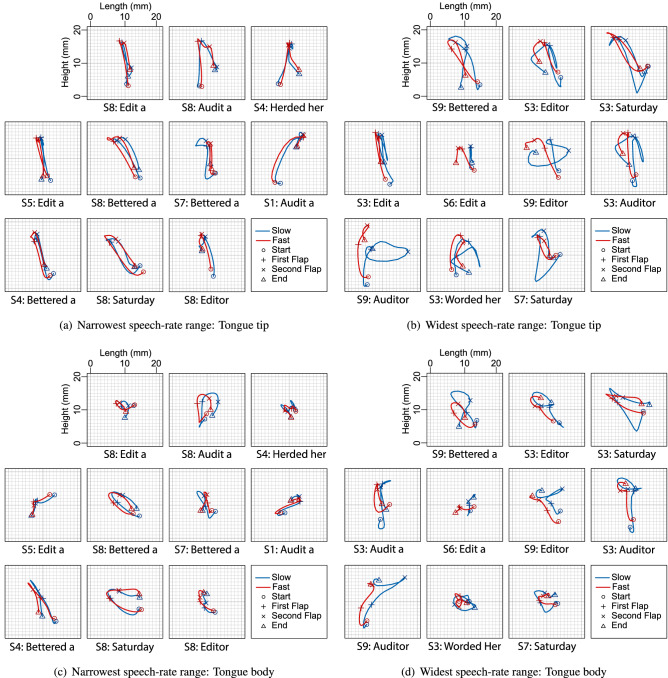
Averaged tongue tip (top left and right) and tongue body (bottom left and right) motion patterns comparing the 10 participants/token types with the narrowest tongue-front displacement ranges (left) with the 10 participants/token types with the widest tongue-front displacement ranges (right), as identified in Fig. 5. Each grid-box shows tongue motion in response to reiterant speech at 3 syllables/s in blue (labelled slow in the legend), and 7 syllables/s in red (labelled fast in the legend).

The information in Fig. 5 allows us to present a visual comparison of tongue and lip motion for the ten narrowest and ten widest tongue-front displacement ranges, as shown to either side of the black dashed lines in Fig. 5. These are separated by articulator such that the tongue tip is shown in Fig. 6a,b, the tongue body is shown in Fig. 6c,d.

Examining individual participant- and token type-specific results shows that participants with a wider speech-rate range exhibit a variety of slow-gait strategies, as illustrated with the blue traces in Fig. 6b,d. Some of these token types show greatly extended tongue-tip motion ranges for the middle vowel (top middle: S3: ‘editor’ and middle left: S3: ‘edit a’), different directions of motion and wider paths (top right: S3: ‘Saturday’ and bottom right: S7: ‘Saturday’), or completely different patterns of motion (bottom right, S9: ‘auditor’ and middle second-left, S9:‘editor’). Similarly varied patterns show through in the tongue body images of Fig. 6c,d. In contrast, all of the fast-gait strategies throughout the red traces in Fig. 6, as well as all of the examples for the narrow speech-rate range shown in Fig. 6a,c, are more similar to each other. This data therefore allows us to diagnose examples of unambiguous gait-change-like behaviour for speakers with very high speech-rate ranges, and unambiguous lack of gait-change-like behaviour for speakers with very low speech-rate ranges.

### Analysis: critical fluctuations

**Figure 7:**
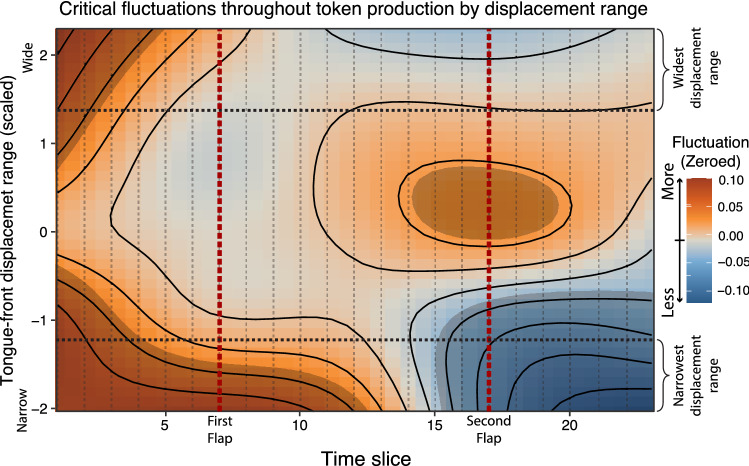
Comparison of critical fluctuations throughout the time course of token production, comparing speakers producing token types based on tongue-front displacement range. The x-axis is divided into fluctuation (F) time slices, which represent the time-course of token production over the contents of 7 Procrustean time slices. The y-axis is the scaled tongue-front displacement range for each speaker and token type. The z-dimension, shown as orange-blue diverging gradient, includes Fluctuation data for each fluctuation time slice. The two red dashed lines show the time slices centered around the first and second flap, referencing Fig. 1. The lower dividing line shows speakers producing token types with the narrowest tongue-front displacement range, corresponding to the motion patterns shown in Fig. 6a,c. The upper dividing line shows speakers producing token types with the widest tongue-front displacement range, corresponding to the motion patterns shown in Fig. 6b,d. Note that this figure shows tongue-front displacement range on the y-axis instead of the x-axis as it is shown in Fig. 5. This was done so that the time slices could match the orientation seen in Fig. 1.

Generalized additive mixed-effects model analysis of critical fluctuations during the time course of token production by tongue-front displacement range are shown in Fig. 7. The model shows that speakers producing tokens with the lowest tongue-front displacement ranges have relatively higher critical fluctuations in the early part of their token productions, spanning from the first vowel through the first flap into the middle of the second vowel. In contrast, they show much lower rates of critical fluctuation from the second half of the second vowel, through the second flap to the end of the third vowel. This constitutes evidence of end-state comfort effects for speakers producing token types with narrow tongue-front displacement ranges.

For speakers producing token types in the middle of the group, there are no end-state comfort effects, but instead the most effort made during the second flap. For speakers with very wide tongue-front displacement ranges, there is again statistically significant evidence for end-state comfort effects, with extra beginning-state effort for the initial vowel. These are the same speakers producing token types that demonstrate two categorically different patterns of motion—one for slow speech, and one for fast speech.

Figure 7 shows the regions of significance for the GAMM whose model output is shown in Table 3. These results show that all of the model parameters are significant, and most importantly that the tensor field shown in Fig. 7 accounts for a significant portion of the variance of the data. This includes the fixed-effect tensor relating tongue-front displacement range and critical fluctuation along time slices, as well as the random-effects for participant, token type, and reiterant speech rate. The entire GAMM accounts for an adjusted $r^{2}$ of 0.452, explaining 47% of the deviance in critical fluctuations in this dataset.

**Table 3:** Results: generalized linear mixed-effects model (Eq. 7).

	edf	Ref.df	F	p-value
*te(FTS, TFd)*	20.04	20.93	12.309	< 0.001
*s(FTS, TFd, Participant, bs = “fs”, m = 1)*	216.31	327.00	9.112	< 0.001
*s(SPS, Participant, bs = “re”)*	31.79	54.00	2.111	< 0.001
*s(Token type, Participant, bs = “re”)*	58.62	86.00	3.427	< 0.001

## Discussion

The results of our research show that as speakers widen their tongue-front displacement range, they gain access to wider speech-rate ranges. At the widest tongue-front displacement ranges, speakers also tend to produce categorically different patterns for their slowest and fastest speech. The speech at the two extremes reveals the most planning, as evidenced through end-state-comfort effects (see Fig. 7). The speakers with the narrowest tongue-front displacement ranges show one stable gait pattern (see Fig. 6a,c. The speakers with the widest tongue-front displacement ranges show two stable gait patterns (see Fig. 6b,d.

Speakers producing token types with the narrowest tongue-front displacement ranges also display very narrow speech-rate ranges. These speakers, producing these token types, use one stable gait. Whether asked to read tokens slowly or quickly, they tend to read those token types at similarly quick rates, and with similar tongue motion patterns. They simply do not speak slowly. Speakers producing these token types also demonstrate strong end-state comfort effects, as seen in the higher rates of critical fluctuations for the first vowel and flap as compared to the lower rates of critical fluctuations for the second flap and final vowel (see the bottom of Fig. 7). These results in these cases show only one pattern of motion produced by following a well-established motor plan (see^40,41^).

In contrast, speakers producing token types with middling tongue-front displacement ranges display middling speech-rate ranges for those token types. These speakers still have one gait, but they stretch and alter the motion to achieve slower and faster speech rates. In these cases, speakers demonstrate no end-state comfort, and instead put significantly more effort into the production of the second flap contact, as seen in higher rates of critical fluctuation around that flap contact (se the middle of Fig. 7). Beginning-state comfort effects are typically taken as examples of reduced motor planning effort^55^ or a lack of experience with the task^40,41^. These results show a range of motion patterns that involve less well-established motor plans.

Lastly, speakers producing token types with the widest tongue-front displacement ranges display the widest speech-rate ranges for those token types. These speakers demonstrate two gaits for these token types. They respond to reiterant speech with the best ability to mimic those speech rates, producing slow speech the slowest, and even producing fast speech the fastest. In these cases, like the speakers producing token types with the narrowest tongue-front displacement ranges, they put the most effort into the beginning of the sequences, demonstrating end-state comfort effects. This result is true for all of the speech rates at which they produce these token types. However, we also know from Fig. 6b,d that these speakers producing these token types often have two categorically distinct patterns of speech—one for slow speech and another for fast speech. These speakers producing these token types have two reasonably well-established motor plans, one for slow speech, and one for fast speech, with fluctuations in between.

These results demonstrate rate-dependent gait changes in movement patterns, leading to an increased movement-rate range, occurring in a non-innate, non-locomotive, and non-rigid motor control system. Specifically, we observed a conflict between the task of mimicking varying speech rates and mechanical limitations on speakers’ tongue movement. Just as fast walkers cannot move as fast as runners, speakers who use a single gait pattern tend to have narrower speech-rate ranges; these ‘one-trick ponies’ restrict their tongue-front motion to movement roughly following a single curve. In contrast, speakers who have greater differences in tongue-front displacement ranges between slow and fast speech appear to gain access to wider speech-rate ranges. Because of the highly individual nature of the variation we observe, we interpret these strategies as emergent rather than neurally pre-determined.

With this evidence, we can argue that widening motion displacement ranges can lead to widening overall motion-rate ranges. A motor system’s exploration of such options may lead that system to develop multiple stable patterns of motion in order to further expand motion-rate ranges. That is, we suggest that such emergent patterns are a necessary part of optimizing rate-varying behaviour in any movement system. This emergent behaviour can then lead to establishment of multiple neurally constructed gait-like options for different motion rates. Given enough evolutionary time, we may expect a motor system to change physical structure over many generations if relying on multiple gaits to expand motion-rate range sufficiently improves fitness of that system. An analogy can be found in soft robotics, where the shape and motion patterns of soft robots^56^ may be simultaneously optimized using emergent evolutionary programming techniques^57^. The complex interactions of each part of the speech system provide a viable mechanism for solving the ill-defined problems of rate-varying behaviour in movement systems. As a result of the interaction of emergent behaviour and experience, we expect that categorical movement-rate based motion solutions may emerge in any motor system for any sufficiently unconstrained task, providing the system with access to wider movement-rate ranges.

## Data Availability

All the supplementary information are available at https://osf.io/7k4ja/?view_only=83ab3adeb363481795ba97e2b481cd9e. This includes 1) source data, 2) source code used to compute the equations, run the statistical models, and produce the images, 3) images of each individual trace, and 4) images of trace averages per participant, token type, and reiterant speech rate.
